# Frontiers in Nanotoxicology

**DOI:** 10.3390/nano12183219

**Published:** 2022-09-16

**Authors:** Alexander A. Gusev

**Affiliations:** 1 Institute for Environmental Science and Biotechnology, Derzhavin Tambov State University, 392020 Tambov, Russia; nanosecurity@mail.ru; Tel.: +7-910-756-4546; 2 Department of Functional Nanosystems and High-Temperature Materials, National University of Science and Technology ”MISIS”, 119991 Moscow, Russia

The Special Issue of *Nanomaterials* “Frontiers in Nanotoxicology” highlights the modern problems of nanotoxicology and nanobiomedicine, including the toxicity of metal-based, silicon-based, carbon-based, and other types of nanoparticles, occupational safety of nanoproduction workers, comprehensive assessment on new biomedical nanomaterials, improvement of nanotoxicology methods, as well as the current state and prospects of research in the fields of theoretical, experimental, and toxicological aspects of the prospective biomedical application of functionalized magnetic nanoparticles activated by a low-frequency non-heating alternating magnetic field, biomedical applications and the toxicity of graphene nanoribbons, and fetotoxicity of nanoparticles. 

In vitro studies included the assessment of caffeic acid lipid nanoparticulate systems on Franz cells associated with the nylon membrane [1], the investigation of the toxicity of 2D nano-layered material ZrS_3_ toward photoluminescent *E. coli* bacteria in a bioluminescence test [2], the toxicity assessment of carbon, silicon, and metal-based nanoparticles on spermatozoa activity, egg fertilization, and early stage of embryo development of the sea urchin *Strongylocentrotus intermedius* [3], and the evaluation of biochemical effects caused by the influence of different types of carbon nanotubes, carbon nanofibers, and silica nanotubes on four marine microalgae species [4]. The contribution of these studies to the development of such areas as percutaneous nanosystems for the delivery of antioxidants, antibacterial nanomaterials, and marine nanotoxicology is obvious. In addition to insight into the mechanisms of cytocompatibility and cytotoxicity of nanoparticles, the authors proposed sensitive and stable in vitro models for future studies.

Using a model eukaryotic organism, i.e., yeast cells, the authors developed improved quantitative nanostructure–activity relationship models for silver nanoparticle toxicity evaluation; new relevant descriptors include the charge of particles, their colloidal stability and ζ-potential, and the ability to generate Ag^+^ ions [5]. Thus, using an original approach to the theoretical prediction of the toxicity of silver nanoparticles, the authors improved the methodology of predictive nanotoxicology.

The authors of in vivo studies focused on the entero- and hepatotoxicity of silver nanoparticles to CBF1 mice [6], assessment of the rat’s immune function after the oral administration of SiO_2_ nanoparticles [7], as well as examination of the ability of the natural-mineral-based novel nanomaterial IFMC to induce an increase of intravascular NO, vasodilation, and the consequent increase of blood flow rate and temperature in a living body of rats [8]. These works have improved our understanding of the mechanisms of the reaction of the whole body of model animals to the impact of nanoparticles which are already used or promising in biomedicine.

In a three-year study of occupational safety of workers in the production of nanocomposites, biomarkers of oxidative stress associated with aerosol nanoparticles exposure were identified and analyzed [9]. As a result, the authors proposed sensitive biomarkers contained in biological fluids for biomonitoring of oxidative stress arising from engineered nanomaterials.

Our Special Issue also features some high-quality review articles. The authors described the recent advances in biomedical applications and the toxicity of graphene nanoribbons including comparisons with such analogues as graphene oxide [10], highlighted prospects and the safety aspects of the biomedical applications of functionalized magnetic nanoparticles activated by a low-frequency non-heating alternating magnetic field [11], and reported the recent updates in the fetotoxicity of nanoparticles, discussing possible causes and mechanisms [12]. These review articles have contributed to evaluating the benefits, uncertainties, and limitations of new nanomaterials and nanotechnological approaches for biomedical applications.

The results and findings are expected to be useful for researchers who are working in the field of nanotoxicology and related areas. Finally, I would like to express my sincere gratitude to all authors who contributed their innovative research to this Special Issue.
